# Presence and Germination of the Probiotic *Bacillus subtilis* DE111^®^ in the Human Small Intestinal Tract: A Randomized, Crossover, Double-Blind, and Placebo-Controlled Study

**DOI:** 10.3389/fmicb.2021.715863

**Published:** 2021-08-02

**Authors:** Joan Colom, Daniela Freitas, Annie Simon, Andre Brodkorb, Martin Buckley, John Deaton, Alison M. Winger

**Affiliations:** ^1^Deerland Probiotics and Enzymes, Food Science Building, University College Cork, Cork, Ireland; ^2^Teagasc Food Research Centre, Moorepark, Fermoy, Ireland; ^3^Mercy University Hospital, Grenville PI, Cork, Ireland; ^4^Deerland Probiotics and Enzymes, Kennesaw, GA, United States

**Keywords:** probiotic, *Bacillus subtilis*, DE111^®^, germination, small intestine, ileostomy

## Abstract

Spore-based probiotics offer important advantages over other probiotics as they can survive the harsh gastric conditions of the stomach and bile salts in the small intestine, ultimately germinating in the digestive tract. A novel clinical trial in 11 ileostomy participants was conducted to directly investigate the presence and germination of the probiotic strain *Bacillus subtilis* DE111^®^ in the small intestine. Three hours following ingestion of DE111^®^, *B. subtilis* spores (6.4 × 10^4^ ± 1.3 × 10^5^ CFU/g effluent dry weight) and vegetative cells (4.7 × 10^4^ ± 1.1 × 10^5^ CFU/g effluent dry weight) began to appear in the ileum effluent. Six hours after ingestion, spore concentration increased to 9.7 × 10^7^ ± 8.1 × 10^7^ CFU/g and remained constant to the final time point of 8 h. Vegetative cells reached a concentration of 7.3 × 10^7^ ± 1.4 × 10^8^ CFU/g at 7 h following ingestion. These results reveal orally ingested *B. subtilis* DE111^®^ spores are able to remain viable during transit through the stomach and germinate in the small intestine of humans within 3 h of ingestion.

## Introduction

The predominant probiotic species on the market are strains of *Lactobacillus*, *Bifidobacterium*, and *Saccharomyces*. However, there is increasing interest in the use of a number of different *Bacillus* species as safe and effective probiotics for humans (Bader et al., 2012; Suva et al., 2016; Lefevre et al., 2017; Anaya-Loyola et al., 2019; Maher, 2019). In order to be efficacious, probiotics need to reach their target location and remain viable. With gastric pH ranging between near-neutral levels immediately after a meal and pH 1 to 2.5 sometime after meal consumption and in the fasted state (Dressman et al., 1990; Kalantzi et al., 2006), the stomach presents a significant barrier for most probiotics (Evans et al., 1988). *Bacillus* is spore-forming bacteria and confers many advantages over the lactic acid bacteria probiotic strains. In their spore form, they are able to survive the harsh gastric environment and reach the small intestine alive. The small intestine lies between the stomach and the large intestine. It is approximately 20 ft (6 m) in length and is responsible for around 90% of digestion and absorption of nutrients from the diet (Korelitz and Janowitz, 1957; Aidy et al., 2015). There are three regions to the small intestine; the duodenum, the jejunum, and the ileum. In the duodenum, secretion of enzymes, bile salts, and bicarbonate allow for neutralization of pH and the digestion of carbohydrates, proteins, and lipids. The jejunum, which follows, is specialized for the absorption of the digested particles. The ileum absorbs the remaining nutrients, particularly vitamin B12 and bile salts, allowing the body to recycle them. In addition to digestion and nutrient absorption, the intestinal epithelium acts as the first barrier against external pathogens and plays a central role in the immune response, containing almost 70% of the entire immune system and the majority of immunoglobulin A-producing plasma cells (Vighi et al., 2008; Santaolalla et al., 2011).

Several *Bacillus* species have been reported to show probiotic potential, including *B. subtilis*, *B. coagulans, Bacillus licheniformis* and *Bacillus clausii* (Horosheva et al., 2014; Cuentas et al., 2017; Lakshmi et al., 2017). *Bacillus subtilis* DE111^®^ is a commercially available probiotic that has been shown to support a healthy gut microbiome and to promote digestive and immune health in both adults and children (Cuentas et al., 2017; Maher, 2019; Paytuví-Gallart et al., 2020; Slivnik et al., 2020; Toohey et al., 2020). The beneficial effect of *B. subtilis* has been shown to be 2-fold, in that both the spore and vegetative forms can confer benefits to the host (Huang et al., 2008; Elshaghabee et al., 2017). Spores of *B. subtilis* can themselves modulate the immune response of the host (Huang et al., 2008); however, the full potential of spore-based probiotics can only be achieved if they also germinate and become active vegetative cells in the small intestinal tract. The vegetative form of *B. subtilis* is also able to modulate the immune response and, in addition, secretes enzymes, antioxidants, vitamins, peptides, and antimicrobial compounds, which help balance the gut microbiota and aid digestion (Elshaghabee et al., 2017). Vegetative *B. subtilis* has also been shown to have antiviral properties against avian influenza and adenovirus (Esawy et al., 2011; Starosila et al., 2017). Germination of *B. subtilis* spores is primarily triggered by nutritional signals (Moir and Smith, 1990). Following passage through the stomach, germination of a *B. subtilis* spore-based probiotic is triggered by the rich nutrient environment of the small intestine (Tam et al., 2006). Once in the vegetative form, the probiotic is able to exert its beneficial effects supporting a healthy gastrointestinal tract. The small intestinal tract has a dynamic microbiome with less diversity than that found in the large intestine (Kastl et al., 2020). The microbiota found in this environment quickly adapt to dietary influences and specializes on metabolism of simple carbohydrates, lipids, and bile salts (Kastl et al., 2020). *Bacillus subtilis* is known to have flexibility in its metabolism and is capable of digesting carbohydrates, proteins, and lipids (Liebs et al., 1988; Eymann et al., 2004). The environment of the small intestine is therefore ideal for germination and proliferation of *B. subtilis*.

Confirming the presence of *Bacillus* spores and vegetative cells in the small intestinal tract is challenging. While studies suggest that ingested *Bacillus* spores can germinate in the small intestinal tract of animals (Spinosa et al., 2000; Casula and Cutting, 2002; Leser et al., 2008), this information is lacking in humans. One yet unexplored approach to investigate the fate of probiotics in the human small intestine involves analyzing the ileal effluent of healthy participants with an ileostomy. An ileostomy is a surgical procedure during which the end of the ileum is passed through an opening in the abdomen known as an ileal stoma. Connected to the stoma is an ostomy bag where all the intestinal contents are collected. As needed throughout the day the contents of the bag are emptied.

The aim of this study was to investigate the survivability and germination of the *B. subtilis* DE111^®^ strain of probiotic in the small intestine using a novel methodology involving healthy adults with an ileostomy.

## Materials and Methods

### Study Design

**Figure 1:**
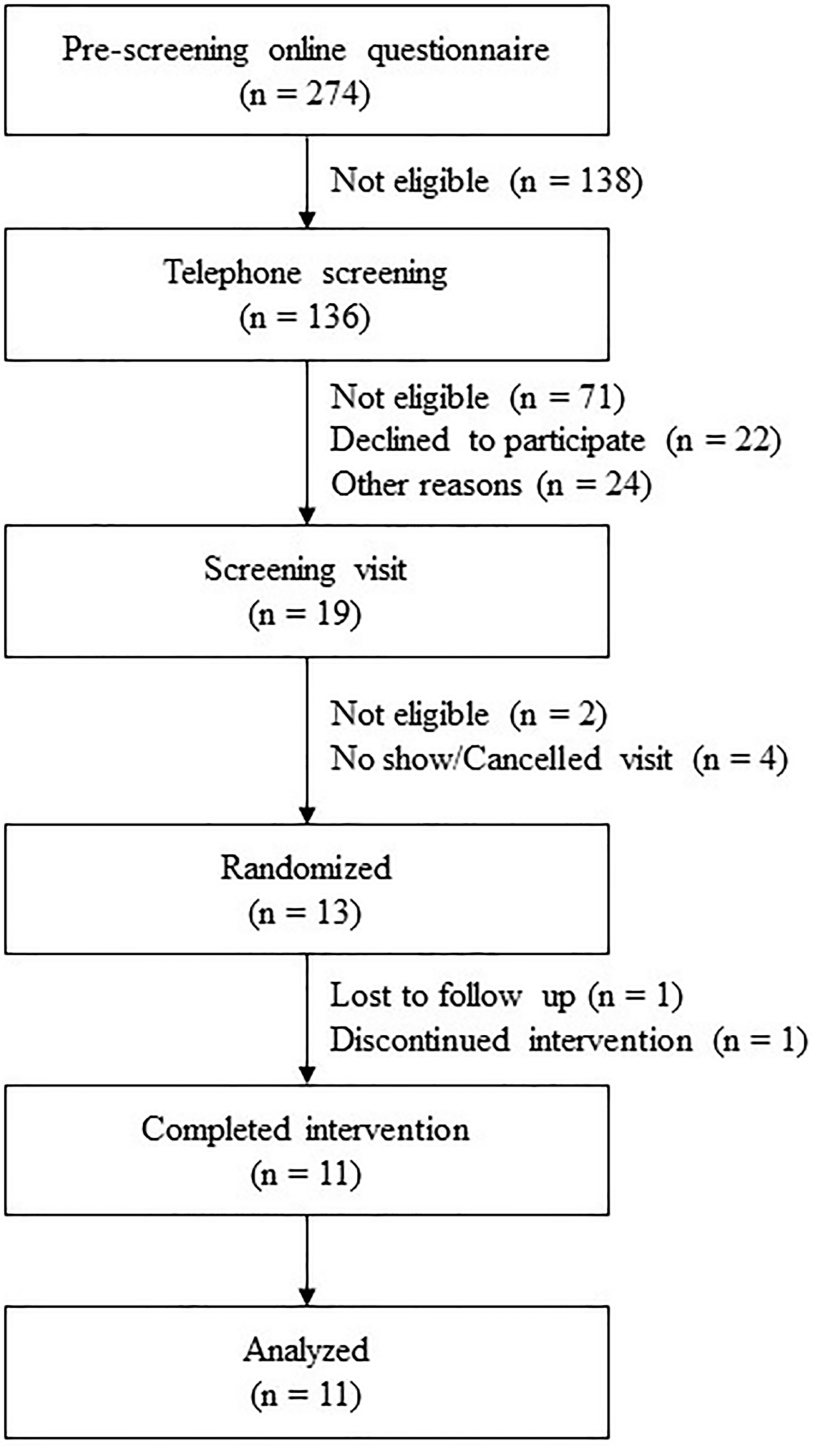
Flowchart of the study profile.

The current study was performed on ileal effluent samples obtained from a wider 4-arm study evaluating the impact of meal properties and dietary supplementation on digestion. This randomized, crossover, double-blind, and placebo-controlled study was carried out between October 2020 and April 2021 at a single site in Ireland. Each of the four interventions was composed of one meal and one study product (placebo or active treatment). The results presented here correspond to two arms only: (1) Meal A + placebo; (2) Meal A + probiotic strain *Bacillus subtilis* DE111^®^. A flowchart of the study is depicted in Figure 1. The protocol was approved by the Clinical Research Ethics Committee of the Cork Teaching Hospitals (review reference number: ECM 4 (d) 05/05/2020) and registered on clinicaltrials.gov (NCT04489810). Adults (aged 18–75) were recruited on the basis of inclusion (having an ileostomy stable for at least 3 months post-operation showing normal stoma function and were otherwise healthy) and exclusion (obstruction of the stoma in the previous 3 months, body mass index below 18 kg/m^2^ or above 30 kg/m^2^, being immunocompromised, history of bariatric surgery, history of drug and/or alcohol abuse, and concurrent participation in other research studies, not using an acceptable method of contraception and not pregnant) criteria. In addition, a current or past diagnosis of one or more of the following was also an exclusion criterion: coeliac disease, allergy to wheat and/or any other ingredients in the study meals; mouth, throat, or active gastrointestinal pathology (other than ileostomy) that may affect normal ingestion and digestion of food; pancreatic disease; and diabetes (both Type 1 and Type 2). Participants were asked not to use any proton pump inhibitors or anti-diarrheal medication during the week and day, respectively, preceding each study day. They were also asked to refrain from excessive alcohol consumption and intensive physical activity the day before the study sessions. All participants gave their written informed consent to participate after receiving oral and written information about the research and study protocol. The study was conducted following the principles of the WMA Declaration of Helsinki and ICH-Good Clinical Practice guidelines.

Eleven participants received either *B. subtilis* DE111^®^ (5 × 10^9^ CFU) or placebo in the morning at breakfast. Each participant received both interventions in this study, in a random sequence and on different days scheduled at least 1 week apart. Participants consumed a standard gluten-free dinner no later than 21h00 the evening before each study session. Only water was allowed after dinner. The following morning, participants arrived at the study site in a fasted state and remained on-site for the duration of the study session. Participants emptied their ileostomy pouch into a sample collection bag prior to consumption of the investigational products and standard breakfast [two pots of porridge (Flahavan’s Organic Original Porridge), one Weetabix, and one glass of water (125 ml)] at 09h00. A standard lunch, at 13h30, consisting of 400 g of a smooth soup (Cully & Sully, Cork, Ireland) and 150 g of jelly (Boyne Valley Group, Louth, Ireland) was consumed by all participants. Throughout the study session, participants’ water intake was monitored but unrestricted (up to 1.5 L each session). Ileal effluent was collected at baseline and once every hour for 8 h after breakfast.

### Study Product

The study products were provided in the form of capsules packaged in identical containers in single servings. The DE111^®^ supplement was composed of *Bacillus subtilis* DE111^®^ (5 × 10^9^ CFU), medium chain triglycerides, and low-moisture rice maltodextrin. The placebo consisted of an identical capsule containing maltodextrin.

### Ileal Effluent Collection and Processing

Ileum effluent sample collection was performed on-site by the participants, who emptied their ileostomy pouches into sterile bags (Buerkle^™^ SteriBag^™^ StandUp Polyethylene Sampling Bags). After sample collection, the participants placed the samples in a polystyrene box with frozen (−80°C) cooling packs prior to on-site processing. Samples were collected every hour for 8 h. Upon collection each sample was weighed, diluted 50:50 (w/w) with phosphate buffered saline (PBS) (7.2–7.4 pH) and thoroughly homogenized by vigorous shaking. Aliquots for bacterial enumeration were stored at −80°C in 40% (v/v) glycerol.

### Dry Weight Determination

Samples (1–7 g) were placed in a sterile 20 ml universal container and placed in an oven at 60°C for 48 h to obtain effluent dry weight. All counts of CFU/g represent the g dry weight of the effluent.

### Enumeration of *Bacillus subtilis*

Mannitol egg yolk polymyxin agar (MYP, Merck) was used as selective medium for detection of the *B. subtilis* DE111^®^ probiotic (Ozkan et al., 2013). Polymyxin B and egg yolk supplements (Merck) were added as recommended by the manufacturer. Colonies of *B. subtilis* DE111^®^ were identified based on morphology, mannitol fermentation (yellow color colonies and surrounding area), and absence of lecithinase activity (lack of white halo around the colonies). This identification was confirmed by 16S sequencing of random colonies isolated from several participants using primers 63f – 5'-CAGGCCTAACACATGCAAGTC-3' and 1387r – 5'-GGGCGGWGTGTACAAGGC-3. Total *B. subtilis* counts were done by performing serial 10-fold dilutions of each sample in PBS. Samples were plated on MYP plates, incubated for 18 h hours at 37°C and colonies counted. To obtain spore counts of *B. subtilis* DE111^®^, an aliquot of each sample was heat shocked by incubating at 75°C for 10 min to inactivate all vegetative cells. Using the effluent dry weights, counts are reported as CFU/g effluent. The number of vegetative cells was calculated using equation 1. Average calculations, including standard deviations, were performed using all participant data points.

Original sample‐Heat shock sample=Vegetative cells (1)

## Results

### Baseline Characteristics of Participants

In total, 274 volunteers were screened for eligibility, of whom 13 were randomized as inclusion criteria were met. One participant was lost to follow-up after the screening visit, another participant dropped out after the first study session. The baseline characteristics of the participants who completed the study are presented in Table 1.

**Table 1:** Baseline characteristics of the participants.

Characteristics	Data
Gender, n (%)
*Female*	4 (36.4%)
*Male*	7 (63.6%)
Age, years	48 ± 14 (24–75)
BMI^1^, kg/m^2^	27.1 ± 3.3 (21.4–29.9)
Fasting blood glucose^2^, mmol/l	5.1 ± 0.7 (3.8–6.8)
Blood pressure (BP), mmHg
*Systolic BP*	126 ± 17 (102–160)
*Diastolic BP*	77 ± 7 (63–87)
Pulse/Heart rate	76 ± 8 (56–90)

*All values reported as mean ± SD [min-max] or absolute number (%)*.

1 *BMI, body mass index*.

2 *Fasting blood glucose based on baseline concentrations on study days*.

### Presence of *Bacillus subtilis* DE111^®^ in the Small Intestine

**Figure 2:**
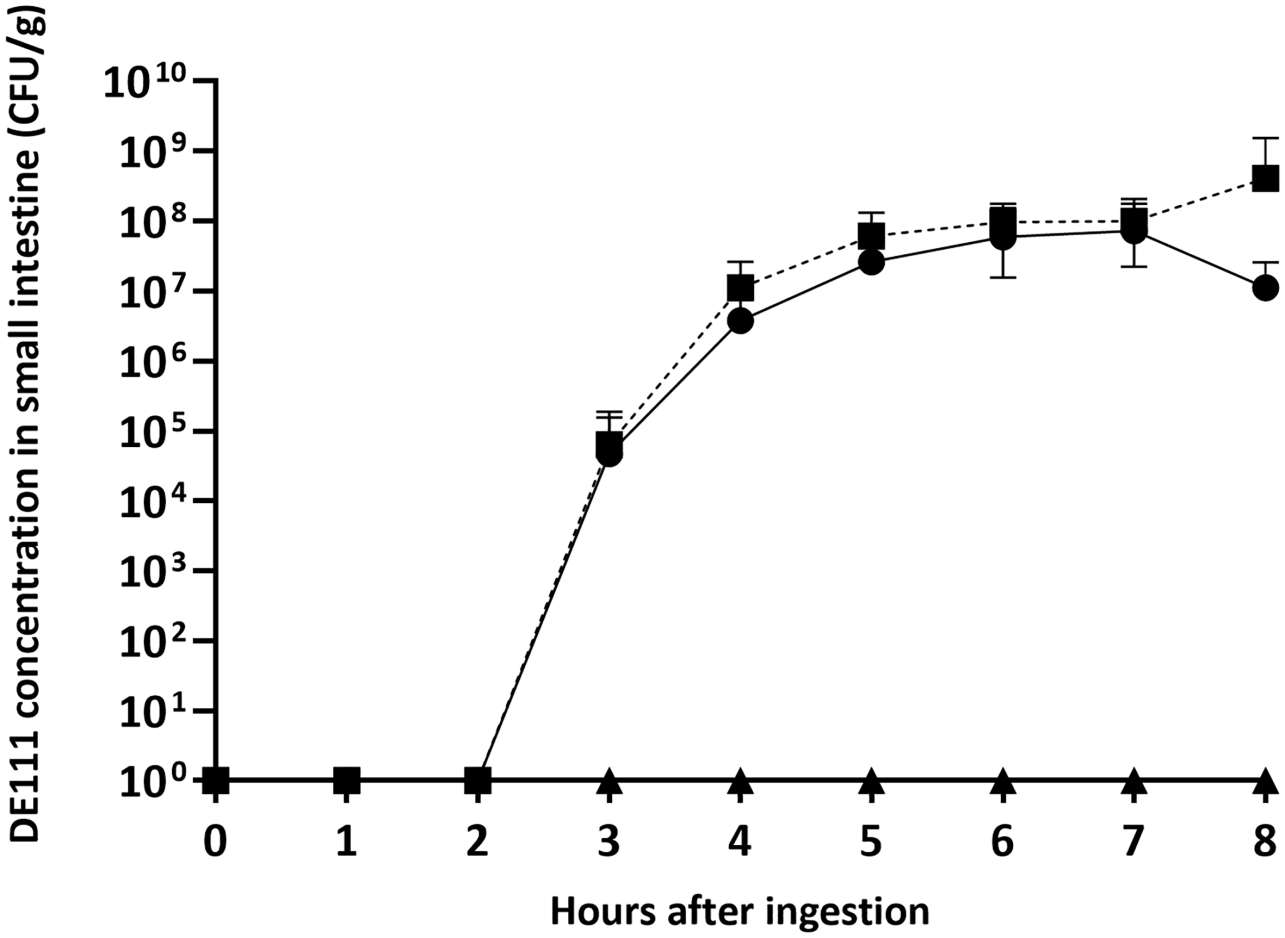
*B. subtilis* DE111^®^ concentration in the small intestinal tract of healthy individuals with an ileostomy stoma. Vegetative DE111^®^ (●), DE111^®^ spores (■), and placebo (▲). Values are average concentrations (*n* = 11) ± standard deviation.

Spores of *B. subtilis* DE111^®^ (6.4 × 10^4^ ± 1.3 × 10^5^ CFU/g) were detected in the small intestinal tract 3 h following ingestion of the probiotic capsule (Figure 2; Supplementary Table 1). An increase in the number of spores over time was seen and reached a peak at 6 h following ingestion (9.7 × 10^7^ ± 8.1 × 10^7^ CFU/g). The same concentration of spores continued to be present in the ileal effluent at each time point assessed until end of the study session at 8 h following ingestion. Over the course of the 8-h study session, a total of 3.0 × 10^9^ ± 6.8 × 10^9^ CFU of the originally inoculated spores were recovered from the small intestinal effluent.

Vegetative cells of *B. subtilis* DE111^®^ were also evident after 3 h (4.7 × 10^4^ ± 1.1 × 10^5^ CFU/g; Figure 2), revealing germination of the spore in the small intestine. Vegetative *B. subtilis* DE111^®^ concentrations in the ileal effluents reached a peak concentration 7 h after ingestion (7.3 × 10^7^ ± 1.4× 10^8^ CFU/g), with the final concentration of 1.2 × 10^7^ ± 1.4 × 10^7^ CFU/g at the final time point.

All participants had both spores and vegetative cells present in their ileal effluent although the rate at which they first presented and persisted varied among individuals (Supplementary Table 1; Table 2). Presence of spores and vegetative cells was seen from 3 h after ingestion, with spores identified in 36% of participants and vegetative cells in 27% of samples at this point (Table 2). Four hours following ingestion, 80% of participants had spores in their ileal effluents and 60% had vegetative cells. All participant samples had spores present 5 h after ingestion and spores remained present in the effluents until the end of the 8-hour study session (Table 2). Detection of vegetative *B. subtilis* DE111^®^ in ileum effluents was 82% after 5 h, 91% at 6 h, and remained similar until the end of the study. All participants had vegetative cells present in their ileal effluent at some time throughout the session (Table 2).

**Table 2:** *Bacillus subtilis* DE111^®^ relative spore and vegetative cell concentration (% of total DE111^®^ counts) in ileal effluents of individual participants (A–K) over the course of the study session (0–8 h).

Hours	Participant											Average abundance
	A	B	C	D	E	F	G	H	I	J	K	
**0**	***0***	***0***	***0***	***0***	***0***	***0***	***0***	***0***	***0***	***0***	***0***	***0 ± 0***
	0	0	0	0	0	0	0	0	0	0	0	0 ± 0
**1**	***0***	***0***	***0***	***0***	***0***	***0***	***0***	***0***	***0***	***0***	***0***	***0 ± 0***
	0	0	0	0	0	0	0	0	0	0	0	0 ± 0
**2**	***0***	***0***	***0***	***0***	***0***	***0***	***0***	***0***	***0***	***0***	***0***	***0 ± 0***
	0	0	0	0	0	0	0	0	0	0	0	0 ± 0
**3**	***13***	***0***	***0***	***35***	***0***	***0***	***0***	***0***	***0***	***53***	***0***	***9 ± 18***
	87	100	0	65	0	0	0	0	0	47	0	27 ± 40
**4**	***0***	***24***	***0***	***28***	***0***	***6***	***29***	***22***	***0***	***NS***	***35***	***20 ± 24***
	100	76	0	72	0	94	71	78	100	NS	65	60 ± 39
**5**	***21***	***67***	***55***	***62***	***16***	***7***	***31***	***0***	***19***	***0***	***27***	***28 ± 24***
	79	33	45	38	84	93	69	100	81	100	73	72 ± 24
**6**	***16***	***42***	***79***	***38***	***38***	***0***	***20***	***3***	***15***	***36***	***44***	***30 ± 23***
	84	58	21	62	62	100	80	97	85	64	56	70 ± 23
**7**	***15***	***73***	***73***	***47***	***28***	***0***	***16***	***2***	***31***	***47***	***8***	***31 ± 26***
	85	27	27	53	72	100	84	98	69	53	92	69 ± 26
**8**	***17***	***0***	***NS***	***59***	***3***	***16***	***44***	***17***	***21***	***0***	***25***	***20 ± 19***
	83	100	NS	41	97	84	56	83	79	100	75	80 ± 19

*NS, no sample available. Average abundance (%) is shown as the average of all participants ± standard deviation. Values in the first row of each cell indicate vegetative counts **(bold italics)**. Values in the second row of each cell indicate spore counts*.

## Discussion

A majority of human intervention studies examining *Bacillus* probiotic behavior in the gut involve samples recovered from the end of the intestinal tract *via* feces (Hanifi et al., 2015). Confirming the presence of *Bacillus* vegetative cells in the small intestinal tract is challenging. To date, germination of *Bacillus* spores in the small intestine of humans has only been characterized in artificial gastrointestinal models (Bernardeau et al., 2017). A majority of studies examining the fate of *Bacillus* spores administered orally have been carried out in animal models and reveal a disparity of results. In one study, mice inoculated with spores of *B. subtilis* and *B. clausii* had no vegetative cells detected in the intestinal tract (Spinosa et al., 2000). In comparison, another mouse model study investigating the spore germination of two different strains of *B. subtilis* revealed the presence of vegetative cells in the jejunum 12–18 h following ingestion (Tam et al., 2006). Molecular approaches based on competitive reverse transcription-PCR, targeting a gene uniquely expressed by vegetative *B. subtilis* cells, detected 1–12% germination of spores in the jejunum and ileum of mice (Casula and Cutting, 2002). In contrast, 70–80% germination of *B. subtilis* and *B. licheniformis* spores was observed in the proximal intestinal tract of pigs (Leser et al., 2008). Taken together, these studies suggest the fate of spore-forming probiotics in the gut is strongly strain dependent and may differ dramatically depending on the model organism used for the study. This highlights the necessity of strain-specific studies that are performed in the target population to collect accurate data regarding the behavior of the probiotic strain in the gut.

This current study is, to the best of the authors knowledge, the first time in which the fate of a probiotic in the small intestine was investigated. A novel clinical intervention trial in healthy human participants with an ileostomy was developed, enabling real-time, direct access to effluent at the end of the ileum (small intestine). Using this method, the ability of the spore-based probiotic *B. subtilis* DE111^®^ to survive gastrointestinal transit and germinate in the small intestine was evaluated. Three hours following the ingestion of a commercially available capsule of *B. subtilis* DE111^®^, spores and vegetative cells were found to be present in the ileum. The number of both spores and vegetative cells increased over the course of 6 h in ileum effluxes and remained constant through to the end of the time course (8 h). The counts in this study are representative of non-adhered cells, with in-situ numbers potentially being higher as *Bacillus* species are known to adhere to intestinal mucus (Elshaghabee et al., 2017) and specifically, DE111^®^ has been seen to adhere to Caco2 cells (unpublished data). While spores of *B. subtilis* have been shown to enhance host immunity in the small intestine (Huang et al., 2008), additional host benefits are only possible if the vegetative form of the bacteria is also present. Detecting vegetative cells of *B. subtilis* DE111^®^ in the small intestinal tract suggest metabolically active bacteria are present, producing key beneficial molecules and supporting a healthy microbiome and gut (Elshaghabee et al., 2017). This finding is significant, as for a probiotic to produce enzymes and small molecules to assist in digestion and exert maximal immune benefits it needs to be in the vegetative form and proliferate in the small intestinal tract (Vighi et al., 2008; Santaolalla et al., 2011; Aidy et al., 2015). The time after ingestion at which vegetative cells were first seen in the small intestinal tract varied between individuals, with a proportion having vegetative cells evident 3 h following ingestion of the probiotic. Widespread presence of vegetative cells across participants was observed after 5 h and remained reasonably constant until the end of the study session. It has been shown that in healthy adults transit time, from ingestion through to the end of the ileum, can range from 157 to 240.5 min (O’Grady et al., 2020). Therefore, the variations observed in the timing of initial presence of *B. subtilis* DE111 in ileum effluxes may be attributed to inherent differences in transit times for each participant. The small intestinal microbiota is dynamic, reflecting the complexity of the environment (Kastl et al., 2020). Recent studies showed daily intake of *B. subtilis* DE111^®^ results in subtle shifts in some key genera, ultimately supporting a healthy microbiome in children aged 2–6 years old (Paytuví-Gallart et al., 2020). Future investigations including metagenomic profiling specifically of the small intestinal microbiota during ingestion of the spore-based probiotic may help further elucidate the beneficial effects of *B. subtilis* DE111^®^ in this region of the gastrointestinal tract.

In conclusion, a unique real-time intervention trial was developed which allowed proof of the survivability of the probiotic *B. subtilis* DE111^®^ through the upper gastrointestinal tract and subsequent germination in the human small intestine. Interestingly, while germination of spores was seen in all participants, the timeline of when vegetative cells first emerged in the ileal effluent was individual dependent. Further studies examining the presence and vegetation of *B. subtilis* over an extended intervention period would be interesting and offer insight into efficacy, metabolic activity, colonization, and re-sporulation of this spore-based probiotic in the small intestinal tract.

## Data Availability Statement

The original contributions presented in the study are included in the article/Supplementary Material, further inquiries can be directed to the corresponding author.

## Ethics Statement

The studies involving human participants were reviewed and approved by the Clinical Research Ethics Committee of the Cork Teaching Hospitals (review reference number: ECM 4 (d) 05/05/2020). The patients/participants provided their written informed consent to participate in this study.

## Author Contributions

DF and AB were involved in protocol writing and study execution. JC and AS performed the sample analysis. MB was the medical advisor for the study and reviewed the manuscript. JC, DF, AB, JD, and AW contributed to the writing of the manuscript. All authors contributed to the article and approved the submitted version.

## Conflict of Interest

JC, AS, JD, and AW are employees of Deerland Probiotics and Enzymes.

The remaining authors declare that the research was conducted in the absence of any commercial or financial relationships that could be construed as a potential conflict of interest.

## Publisher’s Note

All claims expressed in this article are solely those of the authors and do not necessarily represent those of their affiliated organizations, or those of the publisher, the editors and the reviewers. Any product that may be evaluated in this article, or claim that may be made by its manufacturer, is not guaranteed or endorsed by the publisher.
