# Supplementary material for: Presence and Germination of the Probiotic Bacillus subtilis DE111® in the Human Small Intestinal Tract: A Randomized, Crossover, Double-Blind, and Placebo-Controlled Study
Source: Front Microbiol. 2021 Aug 2;12:715863. doi: 10.3389/fmicb.2021.715863 (PMC8366289; doi:10.3389/fmicb.2021.715863)
Supplement: Supplementary file 1 [file Table_1.docx]

**Supplementary Table 1.** *Bacillus subtilis* DE111^®^ spore and vegetative cell concentration (CFU/g) in ileal effluents of individual participants (A-K) over the course of the study session (0-8 hours). Data is presented in DE111^®^ concentration (CFU/g). NS – no sample available. Values in the first row of each cell indicate vegetative counts. Values in the second row of each cell (***bold*** ***italics***) indicate spore counts.

| **Hours** | **DE111^®^ form** | **Participant** | | | | | | | | | | |
| --- | --- | --- | --- | --- | --- | --- | --- | --- | --- | --- | --- | --- |
|  |  | **A** | **B** | **C** | **D** | **E** | **F** | **G** | **H** | **I** | **J** | **K** |
| **0** | Vegetative  ***Spores*** | 0  ***0*** | 0  ***0*** | 0  ***0*** | 0  ***0*** | 0  ***0*** | 0  ***0*** | 0  ***0*** | 0  ***0*** | 0  ***0*** | 0  ***0*** | 0  ***0*** |
| **1** | Vegetative  ***Spores*** | 0  ***0*** | 0  ***0*** | 0  ***0*** | 0  ***0*** | 0  ***0*** | 0  ***0*** | 0  ***0*** | 0  ***0*** | 0  ***0*** | 0  ***0*** | 0  ***0*** |
| **2** | Vegetative  ***Spores*** | 0  ***0*** | 0  ***0*** | 0  ***0*** | 0  ***0*** | 0  ***0*** | 0  ***0*** | 0  ***0*** | 0  ***0*** | 0  ***0*** | 0  ***0*** | 0  ***0*** |
| **3** | Vegetative  ***Spores*** | 8.3x10^3^  ***5.8x10^4^*** | 0  ***1.9x10^4^*** | 0  ***0*** | 1.8x10^5^  ***3.4x10^5^*** | 0  ***0*** | 0  ***0*** | 0  ***0*** | 0  ***0*** | 0  ***0*** | 3.3x10^5^  ***2.9x10^5^*** | 0  ***0*** |
| **4** | Vegetative  ***Spores*** | 0  ***3.2x10^7^*** | 6.5x10^6^  ***2.0x10^6^*** | 0  ***0*** | 4.2x10^6^  ***1.1x10^7^*** | 0  ***0*** | 4.9x10^5^  ***7.7x10^6^*** | 1.7x10^5^  ***4.1x10^5^*** | 3.9x10^6^  ***1.4x10^7^*** | 0  ***6.5x10^5^*** | NS  *NS* | 2.3x10^7^  ***4.3x10^7^*** |
| **5** | Vegetative  ***Spores*** | 5.7x10^7^  ***2.2x10^8^*** | 5.0x10^7^  ***2.5x10^7^*** | 9.6x10^6^  ***8.0x10^6^*** | 7.0x10^7^  ***4.3x10^7^*** | 4.6x10^5^  ***2.4x10^6^*** | 3.6x10^6^  ***4.8x10^7^*** | 3.0x10^7^  ***6.7x10^7^*** | 0  ***3.0x10^7^*** | 1.8x10^6^  ***7.7x10^6^*** | 0  ***4.7x10^7^*** | 6.8x10^7^  ***1.8x10^8^*** |
| **6** | Vegetative  ***Spores*** | 1.9x10^7^  ***1.0x10^8^*** | 2.7*x10^7^*  ***3.7x10^7^*** | 3.1x10^8^  ***8.2x10^7^*** | 1.5x10^8^  ***2.5x10^8^*** | 2.9x10^7^  ***4.8x10^7^*** | 0  ***2.7x10^7^*** | 1.6x10^7^  ***6.6x10^7^*** | 4.2x10^6^  ***1.2x10^8^*** | 4.1x10^7^  ***2.4x10^8^*** | 4.6x10^7^  ***8.3x10^7^*** | 5.5x10^6^  ***7.1x10^6^*** |
| **7** | Vegetative  ***Spores*** | 3.1x10^7^  ***1.7x10^8^*** | 2.0*x10^7^*  **7.5*x10^6^*** | 4.7x10^8^  ***1.7x10^8^*** | 6.6x10^7^  ***7.4x10^7^*** | 9.5x10^7^  ***2.5x10^8^*** | 0  ***1.0x10^7^*** | 1.7x10^7^  ***9.1x10^7^*** | 3.4x10^6^  ***1.6x10^8^*** | 4.5x10^7^  ***1.0x10^8^*** | 5.2x10^7^  ***5.9x10^7^*** | 1.3x10^6^  ***1.5x10^7^*** |
| **8** | Vegetative  ***Spores*** | 6.6x10^6^  ***3.3x10^7^*** | 0  ***6.4x10^6^*** | NS  *NS* | 4.1x10^7^  ***2.9x10^7^*** | 6.9x10^6^  ***2.4x10^8^*** | 1.2x10^6^  ***6.4x10^6^*** | 1.8x10^7^  ***2.3x10^7^*** | 7.0x10^6^  ***3.5x10^7^*** | 3.2x10^7^  ***1.2x10^8^*** | 0  ***3.6x10^9^*** | 2.6x10^5^  ***7.8x10^5^*** |
